# Characterizing patient factors, perioperative interventions, and outcomes associated with inpatients falls after total knee arthroplasty

**DOI:** 10.1186/s43019-024-00215-8

**Published:** 2024-03-08

**Authors:** Kyle W. Lawrence, Lauren Link, Patricia Lavin, Ran Schwarzkopf, Joshua C. Rozell

**Affiliations:** Department of Orthopedic Surgery, NYU Langone Health, 301 East 17th Street, 15th Fl Suite 1518, New York, NY 10003 USA

**Keywords:** Total knee arthroplasty, Fall, Adductor canal block, Tourniquet, Perioperative care

## Abstract

**Introduction:**

Mechanical falls represent a potential adverse event after total knee arthroplasty (TKA) and may introduce further injury and delay postoperative recovery. This study aimed to identify patient characteristics associated with inpatient falls, to determine the impact of inpatient falls on surgical outcomes following TKA, and to describe the relationship between tourniquet and/or adductor canal block (ACB) use and fall rates.

**Methods:**

Patients undergoing primary, elective TKA at a single institution between 2018 and 2022 were retrospectively analyzed. Patients were stratified into groups based on whether they sustained a postoperative inpatient fall or not. Perioperative characteristics, lengths of stay (LOS), rates of 90-day readmissions, and revisions were compared, and fall characteristics were described. Subanalysis was conducted comparing fall incidence based on tourniquet and/or ACB use.

**Results:**

In total 6472 patients were included with 39 (0.6%) sustaining falls. Falls most commonly occurred on postoperative days one (43.6%) and two (30.8%), and were most commonly due to loss of balance (41.9%) or buckling (35.5%). Six (15.4%) fall patients sustained minor injuries, and one (2.6%) sustained major injury (malleolar fracture requiring non-operative orthopaedic management). The LOS (3.0 ± 1.5 vs 2.3 ± 1.5 days, *p* = 0.002) and all-cause revision rates at latest follow-up (10.3% vs. 2.0%, *p* = 0.008) were significantly higher in the fall group. Falls were comparable across subgroups based on tourniquet and/or ACB use (*p* = 0.429).

**Conclusion:**

Patients who fell had a longer LOS and higher revision rate postoperatively. Rates of inpatient falls were comparable regardless of tourniquet and/or ACB use. Concern for inpatient falls should not influence surgeons when considering the use of tourniquets and/or ACBs, though well-designed, large-volume, prospective randomized studies are warranted to better understand this relationship.

## Introduction

Increased focus on enhancing perioperative care and early recovery after total knee arthroplasty (TKA) has been implemented to reduce healthcare costs, improve hospital efficiency, minimize postoperative complications, and optimize patient outcomes [1, 2]. Postoperative falls represent an important, preventable complication following lower extremity orthopaedic procedures, and the risk for sustaining an inpatient fall is greater in patients undergoing TKA who require prolonged hospital admission [3]. Identifying patients at high risk of falling, and implementing fall prevention strategies in the inpatient setting, is critical to mitigating complications [4].

Several patient and postoperative factors, including older age, female sex, obesity, postoperative pain, edema, and motor impairment have been shown to increase fall risk after TKA [3, 5–7]. Further, the use of femoral nerve blocks (FNBs) for pain control during TKA has been shown to impair quadriceps strength and predispose patients to knee buckling and falls [7–9]. Adductor canal blocks (ACBs) have since been a widely adopted alternative as they primarily target sensory fibers of the saphenous nerve [10–12]. However, the motor nerve to the vastus medialis and anterior branch of the obturator nerve—which innervate the adductor muscles—may also be inhibited with ACBs [13, 14]. Several investigations into ACB use have demonstrated a possible association with fall risk, though these studies have been limited by inadequate control groups receiving no block [15–17]. Similarly, whether the intraoperative use of a tourniquet impairs postoperative functional status remains ill-defined [18–20]. Only one study has investigated the relationship between tourniquet use and postoperative falls, and it also lacked a control group [15]. Importantly, it has also yet to be investigated whether patients sustaining an inpatient fall have higher subsequent postoperative complication and revision rates beyond those sustained in the hospital.

This study was conducted to address several of the knowledge gaps present in the current TKA literature regarding postoperative falls, implications of tourniquet and ACB use, and subsequent postoperative outcomes in patients who sustained an inpatient fall after TKA. The primary aim of this study was to identify the impacts of patient characteristics, tourniquet use, and ACB use on the risk of sustaining an inpatient fall after TKA. The secondary aims were to categorize the circumstances and associated injuries sustained during inpatient falls, and to compare postoperative outcomes based on whether patients did or did not sustain an inpatient fall. It was hypothesized that the use of tourniquets and/or ACBs would be higher in patients who had an inpatient fall, and that patients who sustained an inpatient fall would have a higher rate of revision TKA.

## Methods

### Study design and patient stratification

**Fig. 1:**
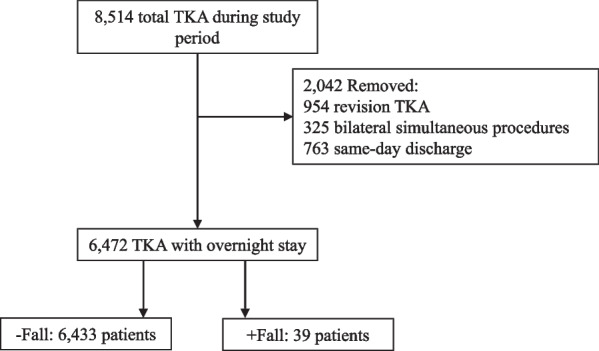
Flow chart of patient inclusion. *TKA* total knee arthroplasty

Prior to initiating any research procedures, this study underwent a formal institutional review board (IRB) review process and all study procedures received approval from the institution’s oversight committee (IRB #i17-1223). This was a retrospective cohort study of all patients age > 18 years undergoing primary, elective TKA at a single, urban, academic orthopaedic specialty care institution with one of 43 arthroplasty surgeons from January 2018 to August 2022. Patients admitted to the hospital for at least one night postoperatively were included in the analysis. Patients undergoing revision TKA, bilateral simultaneous procedures, unicompartmental knee arthroplasty, TKA for fracture, or other non-elective procedures were excluded (Fig. 1). To fully categorize falls for all primary TKA patients within the hospital system, no exclusions were made based on medical or neurologic comorbidities. Additionally, patients discharged on the day of surgery without admission to the hospital were excluded, as these patients carry a distinctly different risk exposure due to their expedited recovery and discharge procedures.

As per hospital protocol and a quality improvement initiative, all perioperative and inpatient falls at the study institution are recorded and stored in a hospital-wide database. The hospital-wide record of patient falls during the study period was reviewed to determine which patients sustained an inpatient fall postoperatively. Patients were stratified into two groups based on whether they did (+ Fall) or did not (− Fall) sustain a fall during their postoperative inpatient hospital stay.

### Baseline patient and perioperative characteristics

Patient baseline characteristics including age, sex, self-identified race, smoking status, and American Society of Anesthesiologists (ASA) class were extracted from the electronic medical record (EMR) and compared between falls groups. Given that medical and neurologic comorbidities could impact fall risk, diagnoses of diabetes with chronic complications, cardiovascular disease (including prior myocardial infarction, heart failure, or peripheral vascular disease), cerebrovascular disease, dementia, and hemiplegia, were also extracted and compared between fall groups. Perioperative characteristics including anesthesia type, ACB use, intraoperative tourniquet use, tourniquet time (minutes), procedure time (minutes), and length of stay (LOS) (days) were extracted from the EMR and compared between falls groups. Inpatient daily opioid consumption was also extracted and converted to morphine milligram equivalents (MMEs) as previously described [21–23] and compared between fall groups. Separate subanalysis of fall incidences across subgroups based on tourniquet and/or ACB use (+ T + B: tourniquet and ACB used, + T− B: tourniquet only used, − T + B: ACB only used, − T− B: no tourniquet or ACB used) was conducted to assess the relationship between falls and the combined use of these perioperative interventions.

### Fall characteristics and postoperative outcomes

For patients who sustained an inpatient fall, characteristics of the fall were described. Fall characteristics included postoperative day of occurrence, reason for fall, whether the fall was assisted or unassisted, and whether an injury was sustained. Reasons for fall included buckling (defined as sudden loss of postural support across the knee joint during weightbearing [24]), loss of balance, unsteadiness, and vasovagal response. Injuries sustained were classified as none, minor, or major. Major injuries necessitated further surgery or a change in postoperative management. Postoperative outcomes, including 90-day readmissions, all-cause revisions at 90 days, 2 years, and latest follow-up, as well as septic and aseptic revisions at latest follow-up, were extracted from the EMR and compared between groups.

### Data analysis

A power analysis was conducted to determine the number of observations required to detect a difference in fall incidences following sub-stratification of patients based on combined ACB and/or tourniquet use. Given that no previous studies have investigated inpatient postoperative fall rates associated with ACBs or tourniquets, fall incidences based on previously reported fall rates after TKA with or without tourniquets/ACBs [3, 7, 16, 25, 26] were used. It was hypothesized that combined tourniquet and ACB use would be associated with a higher fall incidence than either intervention individually, while the use of neither a tourniquet nor an ACB would be associated with a lower fall incidence than either intervention individually or combined. A fall incidence of 1.0% in the − T− B sub-group was anticipated based on previously reported, overall postoperative orthopedic inpatient fall rates [3], and a fall incidence of 3.0% in the + T + B sub-group was hypothesized based on previously reported fall rates with peripheral nerve blockade [7], resulting in a sample size of 822 patients in each of these two sub-groups that would be required to achieve 80% power and a significance level of 0.05.

Fall incidence was calculated as the number of individual patients within the group sustaining at least one inpatient fall (with patients sustaining greater than one fall being counted once), and was represented as count (percentage). Fall occurrence rate was considered as the total number of fall events (including all fall events for patients sustaining greater than one fall) per total number of hospital patient days within the group, and was represented as falls per 1000 patient days. Categorical variables were compared using *chi* squared analyses or Fisher Exact tests, and continuous variables were compared with independent samples t-tests. Categorical variables are presented at count (percentage) and continuous variables are represented as mean (range). Significance was set at a *p* < 0.05. All statistical analyses were performed with IBM SPSS version 28 software (SPSS, Inc, Chicago, Illinois).

## Results

### Study population and fall incidence

In total 8514 TKA procedures occurred during the study period. After removing 954 revision TKAs, 325 bilateral simultaneous procedures, and 763 same-day discharges, 6472 primary TKAs spending at least one night in the hospital remained for analysis, with 6433 in the − Fall group and 39 in the + Fall group (Fig. 1). A total of 40 falls were sustained by the 39 + Fall patients, resulting in an overall fall incidence of 0.6% spanning 14,856 cumulative patient days (fall occurrence rate of 2.7 falls per 1000 patient days). Baseline demographics between groups are demonstrated in Table 1. History of hemiplegia was ubiquitously uncommon but more frequently observed in the + Fall group, though other baseline characteristics were comparable. Mean follow-up time (years) was comparable between groups [+ Fall: 1.4 ± 1.1 (range 1 month to 4 years), − Fall: 1.2 ± 1.1 (range 1 month to 5 years), *p* = 0.355].

**Table 1:** Baseline characteristics for inpatient fall (+ Fall) and no fall (− Fall) groups

Baseline demographics	− Fall	+ Fall	*p*-Value
Age	66.7 ± 9.4	69.3 ± 9.4	0.077
BMI (kg/m^2^)	32.8 ± 6.6	33.1 ± 6.1	0.805
Sex			0.084
Male	2003 (31.1%)	7 (17.9%)	
Female	4430 (68.9%)	32 (82.1%)	
Race			0.484
White	3391 (53.5%)	22 (56.4%)	
Black	1303 (20.5%)	9 (23.1%)	
Asian	283 (4.5%)	3 (7.7%)	
Other	1365 (21.5%)	5 (12.8%)	
Smoking status			0.523
Never	3779 (59.2%)	23 (59%)	
Former	2217 (34.7%)	12 (30.8%)	
Current	387 (6.1%)	4 (10.3%)	
ASA class			0.534
I	165 (2.6%)	0 (0.0%)	
II	3732 (58%)	21 (53.8%)	
III	2467 (38.3%)	17 (43.6%)	
IV	69 (1.1%)	1 (2.6%)	
Comorbidities			
Diabetes + chronic complications	177 (2.8%)	1 (2.6%)	0.943
Cardiovascular disease	697 (10.8%)	5 (12.8%)	0.691
Cerebrovascular disease	539 (8.4%)	5 (12.8%)	0.319
Dementia	28 (0.4%)	0 (0.0%)	0.680
Hemiplegia	11 (0.2%)	1 (2.6%)	**< 0.001**

Statistical significance is denoted with bold text

*BMI* body mass index, *ASA* American Society of Anesthesiologists

### Perioperative characteristics

Perioperative characteristics are demonstrated in Table 2. Compared to the − Fall group, a trend towards higher use of tourniquets [+ Fall: 28 (71.8%) vs − Fall: 4256 (66.2%), *p* = 0.502] and ACBs [+ Fall: 23 (59.0%) vs − Fall: 3034 (47.2%), *p* = 0.150] was observed in the + Fall group. Opioid consumption was significantly higher in the − Fall group on postoperative day zero (*p* = 0.001). There were no significant differences in fall incidences across combined tourniquet/ACB sub-groups (*p* = 0.429) (Table 3). Patients sustaining an inpatient fall had significantly longer LOS than the − Fall group (*p* = 0.002).

**Table 2:** Perioperative characteristics for inpatient fall (+ Fall) and no fall (− Fall) groups

Perioperative characteristics	− Fall	+ Fall	*p*-Value
Anesthesia type			1.00
Regional	5913 (91.9%)	36 (92.3%)	
General	518 (8.1%)	3 (7.7%)	
ACB use	3034 (47.2%)	23 (59%)	0.150
Tourniquet use	4256 (66.2%)	28 (71.8%)	0.502
Tourniquet time (min)	69.7 ± 26.8	60.9 ± 23.9	0.081
Procedure time (min)	105.6 ± 31.8	104.5 ± 20.9	0.831
Daily opioid consumption (MME)			
POD 0	16.2 ± 16.4	9.8 ± 6.8	**0.001**
POD 1	15.2 ± 13.7	12.3 ± 6.7	0.409
POD 2	15.0 ± 11.5	11.3 ± 6.9	0.259
LOS (days)	2.3 ± 1.5	3.0 ± 1.5	**0.002**

Statistical significance is denoted with bold text

*ACB* adductor canal block, *MME* morphine milligram equivalents, *POD* postoperative day, *LOS* length of stay

**Table 3:** Sub-analysis of inpatient fall incidences based on tourniquet (T) and/or adductor canal block (B) use

	− T − B *N* = 1123	+ T− B *N* = 2292	− T + B *N* = 1065	+ T + B *N* = 1992	*p*-Value
Fall Incidence	4 (0.4%)	12 (0.5%)	7 (0.7%)	16 (0.8%)	0.429

### Fall characteristics

Falls were most likely on postoperative day one, were most commonly due to loss of balance, and were predominantly unassisted (Table 4). Six patients sustained minor injuries including pain, edema and ecchymosis, and an upper extremity abrasion. One patient (2.6%) sustained a major injury (ipsilateral medial malleolus fracture treated non-operatively without further complication). No patients sustained wound complications or traumatic arthrotomy secondary to their inpatient fall.

**Table 4:** Fall characteristics among the + Fall group

Fall characteristics	
Postoperative day	
0	6 (15.4%)
1	17 (43.6%)
2	12 (30.8%)
3	3 (7.7%)
> 3	1 (2.6%)
Reason for Fall	
Buckling	11 (35.5%)
Loss balance	13 (41.9%)
Unsteady	4 (12.9%)
Vasovagal	3 (9.7%)
Assisted	
Not assisted	25 (64.1%)
Assisted	14 (35.9%)
Injury sustained	
None	32 (82.1%)
Minor	6 (15.4%)
Major	1 (2.6%)

### Postoperative outcomes

Postoperative outcomes are demonstrated in Table 5. At latest follow-up, patients in the + Fall group had significantly higher rates of all-cause (+ Fall: 10.3%, − Fall: 2.0%, *p* = 0.008) and aseptic (+ Fall: 10.3%, − Fall: 1.5%, *p* = 0.003) revisions. Among the + Fall group, one patient required revision for periprosthetic fracture of the femur secondary to a knee twisting event three weeks postoperatively, one patient was indicated for traumatic tibial component loosening secondary to a fall 1.2 years postoperatively, and two patients were indicated for atraumatic implant loosening and instability at 2.3 and 3.5 years postoperatively. Among − Fall patients, revisions were most commonly indicated for infection (35/131, 26.7%), aseptic loosening (34/131, 26.0%), and arthrofibrosis (18/131, 13.7%).

**Table 5:** Postoperative outcomes for inpatient fall (+ Fall) and no fall (− Fall) groups

Postoperative outcomes	− Fall	+ Fall	*p*-Value
Readmission (90 days)	177 (2.8%)	2 (5.1%)	0.294
All-cause revision			
90 days	10 (0.2%)	1 (2.6%)	0.064
2 years	103 (1.6%)	2 (5.1%)	0.131
Latest follow-up	131 (2%)	4 (10.3%)	**0.008**
Septic revision (latest follow-up)	35 (0.5%)	0 (0.0%)	1.000
Aseptic revision (latest follow-up)	96 (1.5%)	4 (10.3%)	**0.003**

Statistical significance is denoted with bold text

## Discussion

There is a current lack of evidence examining perioperative risks and postoperative outcomes for patients who did and did not sustain an inpatient fall after TKA. This study reports the following key findings: (1) the overall inpatient fall occurrence rate was low (2.7 falls per 1000 patient days), (2) falls most commonly occurred secondary to loss of balance and buckling and were most frequent on postoperative days one and two, (3) + Fall patients had longer LOS than did the − Fall patients but consumed fewer daily opioids in the hospital, (4) fall rates were comparably low across subgroups based on tourniquet and/or ACB, and (5) patients who sustained an inpatient fall had significantly higher revision rates at latest follow-up.

Multiple studies have examined the impact of peripheral nerve blockade use on lower extremity function [10–12, 16, 27–31], proposing a quadriceps weakening effect associated with ACB use, however many of these studies have been limited by inadequate control groups (i.e. patients not receiving a block) [10–12, 27, 28]. Similar analyses of tourniquets and quadriceps strength have supported a quadriceps weakening effect due to tourniquet use, though the degree to which strength is impacted in the early postoperative (i.e. postoperative days one and two) period was not fully assessed [29, 31]. In addition, whether such decreases in quadriceps strength translate to higher rates of postoperative falls has not been clearly established [15–17]. To address this, this study subdivided the patient population into four groups based on whether they did or did not receive a tourniquet and/or ACB, and it was observed that fall incidences were comparably low across all four groups. The use of tourniquet and ACB either alone in or in combination was not associated with a higher incidence of falls. Though these findings support the safety of combined tourniquet and ACB use in TKA, given the previously documented mechanisms for quadriceps weakness and fall risk associated with tourniquet and ACB use, larger scale studies with fall rates at longer follow-up are warranted to validate the study’s conclusions.

Regarding fall characteristics, it was observed that falls predominantly occurred on postoperative days one and two, with fewer falls occurring on the day of surgery or after postoperative day three. Paravlic et al. conducted a time-course analysis of quadriceps strength impairment after TKA, demonstrating the greatest impairment up to three days postoperatively [32]. This observation, in conjunction with current efforts to implement early physical therapy after TKA, as well as postoperative deficits in lower extremity proprioception, may explain the peak in fall incidence in this early postoperative period [33, 34]. Further, falls in the current study most commonly occurred secondary to loss of balance and knee buckling, underscoring the roles of proprioception and quadriceps strength in postoperative fall risk. Surprisingly, it was observed that opioid consumption was higher in patients who did not sustain a fall. It is possible that the + Fall group represents a subset of patients in which both postoperative pain control was adequate and rehabilitation activity participation and ambulation were not impaired by the side effects of opioid consumption, thus providing more opportunity for a fall event to occur.

With respect to postoperative outcomes after discharge, no differences in 90-day readmission rates based on fall groups were observed. Interestingly, revision rates were significantly higher in the + Fall group at latest follow-up. Whereas patients in the -Fall group most commonly required revisions for infection followed by aseptic loosening, patients who did sustain a fall required revisions for component loosening and periprosthetic fracture. Of the four revisions in the + Fall group, two were indicated secondary to an identifiable, mechanical injury after discharge, while the remaining two were indicated for revision for early (2.3 and 3.5 years postoperatively) atraumatic loosening. There is a current paucity of literature demonstrating a causal relationship between mechanical falls and subsequent aseptic loosening in TKA. However, increased mechanical stresses, especially at the tibial component, are known to portend higher aseptic loosening risk, and this had been demonstrated both in patients with elevated BMI and higher activity levels [35–37]. Though mechanistically distinct from the chronic, elevated biomechanical stresses in obese or active patients, patients who are susceptible to repeated falls postoperatively may experience episodes of acute, high-stress forces across their implants predisposing to aseptic loosening over time. Further, cement mantle quality in cemented TKAs [38] and stably maintained corticocancellous contact for osseointegration in cementless TKAs [39], are essential in the immediate postoperative period to achieve adequate fixation and prevent micromotion that can lead to aseptic loosening. It is possible that acute forces associated with fall events in the immediate postoperative period may introduce small disruptions within the newly formed cement mantle or at the interfaces between bone, cement, and prostheses, thus compromising initial fixation and portending greater risk of aseptic loosening. However, future biomechanical studies are needed to understand this relationship.

The findings of the current study provide several clinically relevant considerations in the perioperative management of TKA patients. First, falls sustained while inpatient may be a harbinger of future fall risk or component failure. Thus, close follow-up and consistent postoperative occupational and physical therapy intervention may be warranted in patients that sustain a postoperative inpatient fall to minimize their risk of further complications. Second, the majority of falls that occurred were unassisted, suggesting that routine inpatient supervision protocols are warranted during postoperative ambulation, especially on postoperative days one and two, when falls are most common. Lastly, assessment of the patient’s home environment and potential hazards should be evaluated perioperatively in order to educate patients appropriately on ways to decrease falls. The strengths of this current study include the evaluation of fall rates among a large volume of patients over an extended time period at a large orthopedic specialty hospital. Further, this study stratified patients based on specific perioperative interventions to evaluate fall risks across treatment groups. Additionally, patients’ fall characteristics were able to be described in detail due to the robust fall monitoring protocol, and surgical outcomes could be assessed in patients who fell at latest follow-up.

## Limitations

This study has several limitations. Due to its retrospective design, this study may inherit selection bias and bias due to loss of follow-up. Further, though this study investigated patients who sustained a fall while inpatient and within the inpatient period, it was not feasible to fully assess the incidence of falls after discharge. In addition, due to the overall low incidence of falls during the study period resulting in group imbalance with the preponderance of patients in the -Fall group, these analyses may have lacked sufficient power to detect modest increases in fall risk associated with patient variables and tourniquet or ACB use. However, given the study’s power analysis to detect differences between combined tourniquet and ACB subgroups and the size of the overall population observed over a 4-year time period, these findings are sufficient to conclude that combined tourniquet and ACB use is not sufficient to increase inpatient fall risk to a substantial degree within the patient population of a large, urban surgery center.

## Conclusion

Patients who fell had a longer LOS and higher revision rate postoperatively. Rates of inpatient falls were comparable regardless of tourniquet and/or ACB use. Concern for inpatient falls should not influence surgeons when considering the use of tourniquets and/or ACBs, though well-designed, large-volume, prospective randomized studies are warranted to better understand this relationship.

## Data Availability

The datasets generated during and/or analyzed during the current study are not publicly available due to patient confidentiality but select, blinded data can be made available from the corresponding author on reasonable request.
